# Bone and Kidney Alterations in Diabetic Akita Mice: Evaluating FGF23 Secretion and Urinary Calcium and Phosphate Excretion

**DOI:** 10.3390/ijms27219989

**Published:** 2026-11-07

**Authors:** Giovanna Castoldi, Ilaria Viganò, Barbara Vergani, Isabella Villa, Silvia Ippolito, Marco Casati, Silvia Galbiati, Daniela Gabellini, Alessia Montorfano, Francesco Giordano, Biagio E. Leone, Giovanni Zatti, Alessandro Rubinacci, Gianpaolo Zerbini

**Affiliations:** 1 Dipartimento di Medicina e Chirurgia, Università degli Studi di Milano-Bicocca, 20900 Monza, Italy; barbara.vergani@unimib.it (B.V.); a.montorfano5@campus.unimib.it (A.M.); f.giordano32@campus.unimib.it (F.G.); biagioeugenio.leone@unimib.it (B.E.L.); giovanni.zatti@unimib.it (G.Z.); 2 Unità Complicanze del Diabete, IRCCS Istituto Scientifico San Raffaele, 20132 Milano, Italy; vigano.ilaria@hsr.it (I.V.); galbiati.silvia@hsr.it (S.G.); gabellini.daniela@hsr.it (D.G.); zerbini.gianpaolo@hsr.it (G.Z.); 3 Laboratorio di Endocrinologia e Metabolismo Osseo, Istituto di Endocrinologia e Scienze Metaboliche, IRCCS Istituto Scientifico San Raffaele, 20132 Milano, Italy; villa.isabella@hsr.it; 4 Laboratorio Analisi Chimico Cliniche, IRCCS San Gerardo dei Tintori, 20900 Monza, Italy; silvia.ippolito@irccs-sangerardo.it (S.I.); marco.casati@irccs-sangerardo.it (M.C.); 5 Clinica Ortopedica, IRCCS San Gerardo dei Tintori, 20900 Monza, Italy; 6 Ospedale San Raffaele-Respighi, 20121 Milano, Italy; rubinacci.alessandro@hsr.it

**Keywords:** diabetic bone disease, Akita mice, FGF 23, urinary calcium and phosphate excretion

## Abstract

A growing body of evidence indicates that bone is a target for diabetic complications. Hyperglycemia is associated with bone fragility and increased risk of fractures. In type 1 diabetes, which typically develops in children and adolescents, bone alterations already occur in the skeleton during the growth processes. In type 1 diabetic Akita mice, we analyzed with a pQCT scan the volumetric bone mineral density (vBMD) changes caused by hyperglycemia starting from 10 to 40 weeks of age. The results showed lower cortical vBMD at 20, 30 and 40 weeks of age, whereas cortical thickness and area were lower compared with control mice at all observation times from late growth (10 weeks of age) to aging (40 weeks of age). Fibroblast growth factor 23 (FGF23), synthetized mainly in osteocytes, is considered a ‘bone hormone’, that is critical to the maintenance of phosphate homeostasis. Diabetic Akita mice showed a persistent increase in the urinary calcium-to-creatinine ratio throughout the experimental period. In the presence of renal histological alterations, the urinary phosphate-to-creatinine ratio varied at different ages; its significantly lower value at 30 weeks of age, compared to wild-type mice, was associated with significantly lower plasma FGF23 levels. This study shows that in the Akita mice, the alterations in bone structure, caused by the high plasma glucose levels, are associated with a modulation of urinary calcium and phosphate excretion over time; the latter was only partially associated with the adjustment of FGF23 secretion.

## 1. Introduction

Clinical studies have associated the increase in circulating fibroblast growth factor 23 (FGF23), a key hormone in the regulation of phosphate homeostasis [1,2], with the onset of type 2 diabetes (T2DM) [3]. FGF23, a 251-amino-acid protein primarily synthetized in osteocytes, binds in the proximal kidney tubule to the FGF receptor (FGFR) and its co-receptor klotho and downregulates the membrane availability of the phosphate type II sodium-phosphate co-transporter, NaPi-IIa, leading to an increase in urinary phosphate excretion and a reduction in plasma phosphate levels [2]. Patients with insulin resistance or type II diabetes show higher plasma levels of FGF23 independent of renal function [4,5,6,7], not indicating only a role for FGF23 as a risk factor for the development of diabetes but also suggesting a pathogenic role for FGF23 in the mechanisms involved in insulin resistance, metabolic syndrome, inflammation and obesity [8,9].

The role of FGF23 in type 1 diabetes mellitus (T1DM) is less studied. It remains unclear whether FGF23 is directly linked to adverse effects on glucose metabolism, renal function or cardiovascular risk, as T1DM does not produce a consistent FGF23 pattern [10,11]. Indeed, a Mendelian randomization analysis did not provide evidence for a causal relationship between FGF23 and diabetic renal complications in T1DM, in contrast to T2DM [12], and in a population analysis, patients with T1DM did not show FGF23 values significantly different from controls [13]. Further studies did not clarify the relationship between FGF23 and T1DM, as serum FGF23 concentrations in individuals with type 1 diabetes and a normal albumin excretion rate were comparable to those of healthy controls, and higher FGF23 levels were only found in individuals with advanced kidney disease [10]. However, in a previous study, FGF23 levels in T1DM patients were positively associated with arterial stiffness even after correction for bone mineral parameters, estimated eGFR and classical cardiovascular risk factors [14]. More recently, in a longitudinal study conducted in adolescents with T1DM, predominantly normoalbuminuric at baseline and with hyperfiltration, higher levels of FGF23 were associated with greater increases in eGFR over time, thus establishing a potential link between FGF23 and kidney function in T1DM [15].

Moreover, the critical presence of essential Klotho proteins in skeletal tissues [16] indicates a direct function of FGF23 in the bone-remodeling process, beyond its hormonal actions on the Vitamin D/PTH axis. Given the complexity of bone remodeling [17,18], the mechanistic link between local FGF23 actions and bone cells remains unclear. Possible roles of FGF23 in bone remodeling include osteoclastogenesis, osteoclast-mediated bone resorption and osteoblastogenesis, although all these pathways remain controversial. Recent findings [19] have, however, clearly confirmed the important role of FGF23 in osteogenic differentiation. Since overexpression of FGF23 inhibits the osteogenic differentiation of hBMSCs (human bone marrow stromal cells), leading to osteoporosis, it is reasonable to hypothesize that increased FGF23 levels in diabetes might suppress osteogenic differentiation, thus contributing to the hallmark bone alterations observed in T1DM.

T1DM typically occurs in children and adolescents [20,21]. It is characterized by insulin deficiency due to the loss of the insulin-producing beta cells of the pancreatic islets, generally caused by T-cell-mediated autoimmune disease [22]. T1DM, when poorly treated, is associated with low bone mass caused by reduced bone formation [23,24], leading to a high fracture risk and impaired fracture healing [23,25,26]. In T1DM bone fragility is recognized as a major complication of the disease [27]. The mechanisms underlying osteopenia and poor fracture healing in T1DM are driven by hyperglycemia, insulin deficiency, AGEs accumulation and chronic kidney disease [28], but mechanisms and factors that could represent a link between bone damage and alterations in diabetes target organs, in particular the kidney, are presently still not completely clarified.

The Akita mouse is a T1DM model caused by a mutation in the *Ins2* gene. This animal is born with normal pancreatic islets and insulin-producing β cells, but the *Ins2* mutation causes abnormal structural folding of insulin, pancreatic beta cell dysfunction and, finally, reduced capacity to secrete insulin [29]. Male Akita mice at 5 weeks of age spontaneously develop diabetes with high blood glucose and hypoinsulinemia and progressively develop proteinuria with renal histological damage, influenced by genetic background [30,31], and alterations in the skeleton as the disease progresses over time [32,33]. Due to these characteristics, the Akita mouse offers the opportunity to study untreated hypoinsulinemic hyperglycemia throughout the entire mouse’s lifespan.

Bone fragility is a common feature of both T1DM and T2DM, two diseases that share similar levels of hyperglycemia but that strongly differ for plasma insulin levels, increased in T2DM and barely detectable in T1DM. This evidence raises the question of whether hyperglycemia, and consequent glucotoxicity, may represent the key regulator of FGF23 metabolic balance in the case of diabetes.

To clarify this issue, we studied the plasma concentration of FGF23, the urinary calcium and phosphate excretion rate and the bone structure in the Ins2Akita mouse at different ages [31], the ideal animal model to study the impact of glucotoxicity on different organs.

In this study we demonstrate that in the Akita mice, the alterations in bone structure caused by high plasma glucose levels are associated with a modulation of urinary calcium and phosphate excretion, only partially driven by the adjustment of FGF23 secretion, in the presence of significant renal histological alterations.

## 2. Results

### 2.1. Animal Characteristics and Serological Parameters

Body weight and non-fasting blood glucose levels were measured in Akita mice and corresponding WT mice at 10, 20, 30 and 40 weeks of age (Figure 1a,b).

At 10 weeks of age, the BW in Akita mice was similar to WT mice at the same age. At 20, 30 and 40 weeks of age, the BW in Akita mice was lower compared to WT mice of the same age, indicating that in Akita mouse growth is compromised due to the glycometabolic imbalance (Figure 1a).

As shown in Figure 1b, Akita mice showed a marked increase in blood glucose levels already detectable at 10 weeks of age, reaching values around 600 mg/dL at 20 weeks of age and stabilizing thereafter. At every timepoint blood glucose levels in Akita mice were significantly higher than in WT mice.

Plasma levels of creatinine and phosphate at 10, 20, 30 and 40 weeks of age in Akita and WT mice are shown in Table 1.

Plasma creatinine values were not different in Akita mice as compared to WT mice of the same age, in line with previous reports showing that Akita mice (like other rodents) are resistant to the development of renal failure [34].

Plasma phosphate values tended to be higher in Akita mice with respect to the corresponding WT mice at 10, 20 and 30 weeks of age. This phenomenon is detectable until 40 weeks of age; from then, a reduction in phosphate values was shown in Akita mice compared to age-matched WT mice (Table 1).

### 2.2. pQCT Analysis of Bone Parameters at 10, 20, 30 and 40 Weeks of Age in Akita Mice

The pQCT measurements of trabecular volumetric bone mineral density (Tb-vBMD) at the tibial metaphysis and of cortical volumetric bone mineral density (Ct-vBMD) at the tibial diaphysis of Akita mice and WT and at 10, 20, 30 and 40 weeks of age are shown in Figure 2a.

No significant differences in Tb-vBMD at the tibial metaphysis were detected between Akita and WT mice at all observation times from 10 to 40 weeks of age (Figure 2a). In Akita mice, Tb-vBMD at 40 weeks of age was significantly lower than at 20 weeks of age. Similarly, in WT mice, Tb-vBMD at 40 weeks of age was significantly lower than at 10 and 20 weeks of age.

In contrast, Ct-vBMD at the tibial diaphysis was significantly lower in Akita mice compared with WT mice at all matched ages (Figure 2a), except at 10 weeks. In Akita mice, Ct-vBMD at 40 weeks was significantly lower than at 10 and 20 weeks of age. A similar pattern was observed at 30 weeks, with Ct-vBMD being lower than at 10 and 20 weeks of age.

To further characterize the bone structure of Akita mice, we also measured by pQCT the size parameters that define bone growth from 10 to 40 weeks of age. Akita mice exhibited significantly lower cortical thickness (Ct-Th) and cortical area (Ct-Ar) compared with wild-type mice at all matched ages from 10 to 40 weeks. A similar trend was observed for the periosteal circumference (Ct-PeriC), but it did not reach statistical significance (Figure 2b). In Akita mice, Ct-Th at 30 and 40 weeks were significantly lower than at 10 and 20 weeks of age. Ct-Ar did not significantly change over time in both genotypes. The CT-PeriC was not significantly different in Akita compared to wild-type mice, although in Akita a trend for lower values was observed at 20 and 40 weeks. Ct-PeriC did not significantly change over time in both genotypes. Ct-endoC showed a different pattern over time. While no significant differences were observed between Akita and WT mice at any matched ages, Ct-endoC values in Akita mice at 30 and 40 weeks were significantly higher than those observed at 10 and 20 weeks of age. A similar pattern was observed in the WT mice, except that Ct-EndoC at 30 weeks did not differ from that at 10 weeks of age (Figure 2b).

The strength strain index (SSI), which represents a surrogate index of bone mechanical competence, was also lower in Akita mice than in wild-type mice at 20, 30 and 40 weeks of age (Figure 3). In WT mice, SSI at 40 weeks was significantly higher than at 10 and 20 weeks of age, whereas it did not improve over time in Akita mice (Figure 3).

### 2.3. Plasma Levels of Fibroblast Growth Factor 23 at 10, 20, 30 and 40 Weeks of Age in Akita Mice

Plasma FGF23 was measured at 10, 20, 30 and 40 weeks of age in Akita mice and corresponding WT mice, as shown in Figure 4.

At 10 and 20 weeks of age, plasma levels of FGF23 were similar in Akita mice and age-matched normoglycemic controls. Conversely, in Akita mice at 30 weeks of age, plasma FGF23 levels were lower with respect to WT mice of the same age. Finally, at 40 weeks of age, plasma FGF23 levels did not differ anymore between Akita and WT mice. Plasma FGF23 levels showed a similar trend of increasing over time, except at 30 weeks of age in Akita mice, in both WT and Akita mice.

### 2.4. Urinary Calcium-to-Creatinine and Phosphate-to-Creatinine Ratio at 20, 30 and 40 Weeks of Age in Akita Mice

Urinary calcium-to-creatinine ratio and phosphate-to-creatinine ratio were measured in adult Akita mice and corresponding WT controls at 20, 30 and 40 weeks of age (Figure 5a,b).

In Akita mice urinary calcium excretion was higher when compared to WT mice (Figure 5a) for the entire period of observation. Interestingly, at 40 weeks of age, with the progression of the disease, urinary calcium excretion remained markedly higher in Akita mice (Figure 5a). Phosphate excretion was significantly lower in Akita mice at 30 weeks of age compared to corresponding WT mice at the same age (Figure 5b).

### 2.5. Kidney Histological Damage at 40 Weeks of Age in Akita Mice

Histological examination of renal sections from 40-week-old Akita mice (C,D) showed an increase in the mesangial matrix as compared to WT mice (A,B) (Figure 6), as evidenced by the positive PAS staining (B,D). Tubular dilation is observed in renal histological sections of the Akita mice (C) as compared to WT mice (A).

The morphometric analysis showed a significant increase in tubulo-interstitial fibrosis, quantified by computerized image analysis after Sirius red staining, in Akita mice as compared to WT mice. No significant differences were observed in glomerular fibrosis (Figure 7a,b).

## 3. Discussion

The results of this study show that the Akita mice develop hyperglycemia-driven bone damage that can be demonstrated and quantified by pQCT. This dysfunction is paralleled by renal histological alterations and is associated with a modulation of urinary calcium and phosphate excretion that is only partially explained by FGF23 secretion.

FGF23 has become an important focus in diabetes research because of its potential influence on glucose metabolism. Although its role as a risk factor and possible pathogenic regulator has been extensively described in T2DM [3], its relevance in T1DM remains controversial.

In T2DM, FGF23 levels may increase through several converging mechanisms, including kidney dysfunction, inflammation, metabolic stress and disordered mineral metabolism. This increase has been associated with insulin resistance, β-cell dysfunction, impaired bone metabolism and cardiovascular risk [3]. In T1DM, as discussed, it remains unclear whether FGF23 is consistently elevated independently of kidney dysfunction and whether it is directly linked to adverse effects on glucose metabolism or on the kidney, bone and cardiovascular systems.

A recent experimental study on streptozotocin (STZ)-treated mice, a T1DM model characterized by toxic disruption of insulin production, suggested that FGF23 may also act as a pathogenic regulator in T1DM [35]. Unlike the STZ-treated mice, in which streptozotocin injection abolishes insulin production, making periodic insulin administration essential to prevent the onset of ketoacidosis [36], the Akita mouse spontaneously maintains minimal insulin production over time, allowing it to survive. Both models develop marked hyperglycemia, but Akita mice show less severe bone loss [37].

At 40 weeks of age—the longest timepoint in our analysis—Akita mice exhibited mild histological alterations in the renal parenchyma, tubules and glomeruli, as well as increased tubulo-interstitial fibrosis (Figure 6 and Figure 7), in the absence of changes in plasma creatinine levels, confirming that the Ins2Akita model (on C57BL/6J background) is resistant to the development of renal failure [30]. Even though we cannot fully exclude that, with time, Akita mice may experience a subtle renal dysfunction (renal function in this study is based on plasma creatinine alone), we reasonably believe that the renal alterations observed do not lead to significant changes in plasma FGF23 levels.

pQCT analysis indicated that Akita mice did not show a consistent deterioration of trabecular bone, namely Tb-vBMD. In contrast, they developed a significant impairment of cortical bone accrual, as demonstrated by significantly lower cortical vBMD, cortical thickness and cortical area (CT-vBMD, CT-Th, Ct-Ar), compared with wild-type mice. A similar trend was observed for the periosteal circumference (Ct-PeriC) at 20 and 40 weeks of age, but it did not reach statistical significance. The SSI index, a surrogate parameter of bone mechanical competence, did not increase progressively in Akita mice with age as it did in WT mice. Conversely, endosteal circumference at the cortical bone, a geometric parameter related to bone resorption activity, and the trabecular vBMD at the metaphysis were not significantly different from wild-type mice.

The FGF23 levels in Akita mice were significantly lower than in WT mice at 30 weeks of age but recovered by 40 weeks of age. At the same evaluation time, phosphate excretion was also temporarily lower in Akita mice than in WT mice. In contrast, urinary calcium excretion, which is known to increase in the presence of diabetes through multiple mechanisms [38,39,40,41,42,43], remains higher in Akita mice than in WT mice during the entire experimental period.

The first finding of our study was the pQCT-based characterization of the bone phenotype in Akita mice, across a broad developmental range, from late growth (10 weeks) to aging (40 weeks). This strategy allowed us to assess bone characteristics when insulin misfolding has already produced established type 1 diabetes (10 weeks); at 20 weeks, when long-term diabetes affects the mature skeleton; and at 30–40 weeks, when chronic diabetes influences the aging bone phenotype.

Our findings are in line with the microCT analysis of Akita mouse femurs at 21 weeks of age [32], which showed a cortical bone deficit with significantly reduced cortical area and thickness in the Akita genotype. These alterations were translated into a lower polar moment of inertia, a gold-standard geometric indicator of structural strength [32]. Our results also indicated that the long-standing diabetic condition further compromised bone health of the Akita mice of older ages. The cortical parameters defining cortical bone status at 30 and 40 weeks of age, cortical density (Figure 2a) and thickness (Figure 2b), were in fact significantly lower than those observed at 10 and 20 weeks of age.

The SSI index used in our study (Figure 3), a surrogate measure of mechanical competence that also incorporates cortical vBMD [44], reproduced the same structural deficit previously reported [32]. Although derived from lower-resolution pQCT scans, it further supported the presence of impaired accrual of physiological cortical bone phenotype in Akita mice. Indeed, SSI became significantly lower in Akita compared to WT mice from maturity (20 weeks of age) to aging (40 weeks of age).

In addition, we found that the bone mass accrual was already partially impaired by 10 weeks of age, as indicated by lower Ct-Th and Ct-Ar values, suggesting an early disruption of normal cortical maturation in Akita mice. Since this deficit was not compensated at skeletal maturity and persisted into later adulthood, it might have been established early and has remained refractory to recovery, as shown by the developing pattern of SSI. The absence of a significant difference in the endosteal perimeter (Ct-EndoC) between Akita and wild-type (WT) mice argued against enhanced endosteal bone resorption as a major contributor to the observed bone phenotype. Of note, the consistently higher Ct-EndoC observed in both Akita and WT mice at 30 and 40 weeks compared with that observed at 10 and 20 weeks of age suggests that the endosteal bone modeling required for the growth-related enlargement of the medullary cavity occurred without detectable differences between the two genotypes. Our results were therefore in agreement with previous findings [32], which also reported no significant difference in medullary area between Akita and WT mice. We did not measure bone turnover markers, which would have been helpful in assessing bone resorption; however, the absence of any increase in CTX-1 in Akita mice, as previously reported [32], was consistent with our pQCT assessment.

Together, all these observations suggest that osteoclast activity, if involved, is unlikely to be the main driver of bone damage in T1DM and that the impaired acquisition of the bone phenotype in Akita mice is primarily driven by defective bone formation. This defect may result from insulin deficiency, sustained hyperglycemia in the extracellular environment and accumulation of advanced glycation end products (AGEs). From the mechanistic point of view, these metabolic derangements might result in impaired WNT signal in bone, mainly due to the increment of sclerostin, a WNT inhibitor. Indeed, when the sclerostin-resistant Lrp5A214V mutation, associated with high bone mass, was introduced in mice carrying the Ins2Akita mutation (Akita), the Akita bone phenotype improved [45].

In our study, the absence of a significant difference in the trabecular volumetric bone mineral density between Akita and wild-type mice is apparently in contrast with the trabecular bone deficit in the Akita mice, previously described at the femoral metaphysis [32]. This discrepancy may partly reflect the lower resolution of pQCT, which could reduce the sensitivity to detect subtle trabecular changes. In fact, the higher Tb-vBMD observed in Akita mice at 20 weeks of age compared with wild-type mice may reflect a partial volume artifact associated with pQCT measurements. However, our results are in line with the post-mortem microCT determination of trabecular volumetric bone density that showed no significant difference at the proximal tibial metaphysis between Akita and wild-type mice at 30 weeks of age ([45], in Supplemental Figure S3). The discrepancy between tibia and femur of the trabecular volumetric density outcome in Akita mice remains therefore unsolved, although it might be possibly related to the site-specific sensitivity of bone to metabolic challenges [46].

In Akita mice, glycosuria develops alongside hyperglycemia and persists throughout the animal’s life [47]. The excess of glucose in the urine is accompanied by an excess of calcium excretion. Consistent with these findings, we observed a significant increase in the calcium-to-creatinine ratio, likely resulting from the persistent hyperglycemia and glycosuria. The mechanistic link between glucose and calcium excretion is complex, and it might be related to the osmotic drawing force of glucose into the tubular lumen, the diabetes-dependent increase in glomerular filtration rate and the involvement of the extracellular Ca2+-sensing receptor and transcellular calcium transporters (TRPV5, calbindin-D28K) [39,40,41].

Being independent of parathyroid hormone [43], it is unlikely that hypercalciuria plays a causative role in the bone phenotype of the Akita mouse. It is also unlikely that the hypercalciuria reflects a bone-resorptive process, as bone resorption, which, as discussed above, does not appear to be primarily involved in this mouse model of T1DM.

Our study focused on FGF23 secretion because of its potential regulatory role in phosphate metabolism. Our data did not show increased plasma FGF23 levels in the Akita mouse, unlike what was observed in the STZ model [35], supporting the hypothesis that FGF23 regulation in T1DM differs from that observed in T2DM due to distinct upstream drivers.

In our experimental condition, the absence of higher plasma FGF23 levels compared with wild-type mice rules out a pathogenetic role for FGF23 in shaping Akita bone phenotype, which remains attributable to FGF23-independent impairment of osteogenesis.

At 30 weeks, when diabetic conditions are fully established, it was interesting to note the concomitant decline in phosphate excretion and FGF23 secretion, consistent with the phosphaturic action of FGF23. However, given the observational design of our study, this association at 30 weeks cannot be attributed to any direct regulatory role of FGF23.

Our study has several limitations. To maximize bone assessment by pQCT and avoid the stress and bias of anesthesia, we performed urinary and plasma measurements at the end of each timepoint and the scans on excised bones, thus analyzing different groups of animals. This approach inevitably increased variance and the likelihood of missing subtle but potentially meaningful pathological changes. Moreover, the relatively small and variable sample sizes, the use of creatinine as the primary indicator of kidney function and the absence of bone turnover markers and additional regulators of phosphate metabolism, such as PTH, vitamin D, Klotho and tubular phosphate transporters, constitute additional limitations of our study, which remained exploratory.

## 4. Materials and Methods

### 4.1. Experimental Protocol

Animal husbandry was in conformity with the Institutional Guidelines in compliance with National laws and policies (D.L.n. 116, Gazzetta Ufficiale della Repubblica Italiana, suppl.40, 18 February 1992). All the experimental procedures were conducted in accordance with the European Community guidelines (Directive 2010/63/EU), the Guide for the Care and Use of Laboratory Animals published by the US National Institutes of Health (NIH Publication No. 85-23, revised 1996) and the San Raffaele Institutional Animal Care and Use Committee (IACUC). Experiments were performed in male Ins2^Akita^ (Akita) mice and C57BL/6J wild-type (WT) mice in a temperature-controlled room with a 12:12 light–dark cycle for the entire experimental period. Mice had free access to standard chow and water. Body weight (BW, g) and blood glucose (mg/dL; Blood Contour TS Glucometer-Bayer) were measured twice a month. For the entire experimental period, the mice did not receive any pharmacological treatment.

At the end of the planned experimental periods (10, 20, 30 and 40 weeks of age), Akita and WT mice (n = 8–10 for each timepoint) were euthanized by cervical dislocation. A blood sample was taken from the tail. Urine was collected directly from the bladder. Non-fasting plasma and urinary creatinine (Cr, mg/dL) and phosphate (P, mg/dL) and urinary calcium (Ca, mg/dL) were measured by a colorimetric technique on Cobas Roche (Mannheim, Germany). Mouse fibroblast growth factor 23 (FGF23) in plasma was measured by an Elisa array (Invitrogen, Thermo Fisher Scientific, Waltham, MA, USA), following the manufacturer’s instructions. Urinary excretion of calcium and phosphate was expressed as a ratio to urinary creatinine. Tibiae were excised and fixed with 10% formalin solution, neutral buffered and used for peripheral quantitative computed tomography. The kidneys were excised, sectioned longitudinally, fixed with 10% formalin, embedded in paraffin and used for light microscopic examination and morphometric analysis to evaluate glomerular and tubulo-interstitial fibrosis [48,49].

### 4.2. Peripheral Quantitative Computed Tomography (pQCT) Analysis

Bone tomographic measurements were obtained at 10, 20, 30 and 40 weeks of age using a Stratec Research SA+ pQCT scanner (Stratec Medizintechnik GmbH, Pforzheim, Germany) with a voxel size of 70 µm and a scan speed of 3 mm/s. Following sacrifice, the excised tibiae were secured in manufacturer-provided holders to maintain alignment of the long axis parallel to the imaging plane. Longitudinal positioning was verified by a “scout scan”. Scans were acquired at the proximal metaphysis and mid-diaphysis. Data were analyzed using pQCT software version 6.00B (contour mode 2, peel mode 2), applying thresholds of 500 mg/cm^3^ for trabecular and total bone and 710 mg/cm^3^ for cortical bone. Trabecular analysis included trabecular volumetric bone mineral density (Tb.vBMD). Cortical analysis included: cortical volumetric bone mineral density (Ct.vBMD); cortical thickness (Ct-Th); cortical area (Ct-Ar); periosteal circumference (Ct-PeriC); endosteal circumference (Ct-EndoC) [50].

### 4.3. Kidney Histology and Histomorphometry

Kidneys from 40-week-old Akita and WT mice underwent histological analysis and measurement of tubulo-interstitial and glomerular fibrosis. Renal transmural sections (4 µm) were deparaffinized, rehydrated and stained with hematoxylin-eosin (H&E) and Periodic acid–Schiff (PAS), following standard techniques. Changes in renal morphology were assessed by light microscopic analysis of sections. For all mice a renal section (4 µm) was also stained with collagen-specific Sirius Red stain and used for morphometric analysis of renal fibrosis, as previously described [48,49]. Scanning images were acquired using a motorized Olympus BX63 microscope equipped with a DP80 camera and software cellSens (https://www.olympus-lifescience.com/en/software/cellsens/, accessed on 28 September 2026) (Shinjuku Monolith, Tokyo, Japan). Images were analyzed for collagen volume fraction in tubulo-interstitial and glomerular areas with computerized imaging software (Qu-Path, v5) [51]. Two pathologists, blinded to the experimental protocol, performed image analysis.

Glomerular and tubulo-interstitial collagen fractions were evaluated at X20 magnification, respectively, in a semi-automated fashion in 15 traced glomeruli (considered as matrix, cells, capillary loops and space surrounding glomerular segments) and in an automated fashion in 10 fields of the same size without vessels or glomeruli, randomly selected from each kidney section. The glomerular collagen fraction was expressed as the ratio of red-stained collagen area to glomerular area; the tubulo-interstitial collagen area was expressed as μm^2^.

### 4.4. Statistical Analysis

Differences among the groups of WT and Akita mice at 10, 20, 30 and 40 weeks of age for body weight, blood glucose, plasma creatinine and phosphate values, pQCT parameters (Tb.vBMD, Ct.vBMD, Ct-Th, Ct.Ar, Ct-PeriC, Ct-EndoC, SSI), plasma FGF23 levels, urinary calcium-to-creatinine ratio and urinary phosphate-to-creatinine ratio were assessed with the use of two-way ANOVA, followed by Bonferroni’s post hoc test. Differences among the means were considered significant at *p* < 0.05. Differences between WT and Akita mice at 40 weeks of age for tubulo-interstitial and glomerular fibrosis were assessed by the Mann–Whitney test. Differences between the means were considered significant at *p* < 0.05.

## 5. Conclusions

Our study has evaluated the interplay among bone mass accrual, kidney function and hyperglicemia in Akita mice by focusing on calcium and phosphate excretion and FGF23 secretion. The bone phenotype of Akita mice has been characterized, particularly for the impairment of cortical volumetric bone density and geometric parameters, with subsequent impact on the development of mechanical competence. Taken together, these findings support the use of Akita mice as a suitable model for investigating the skeletal complications of T1DM. This model provides a valuable experimental framework for the detailed evaluation of bone metabolism and for the future development of targeted therapeutic strategies.

## Figures and Tables

**Figure 1:**
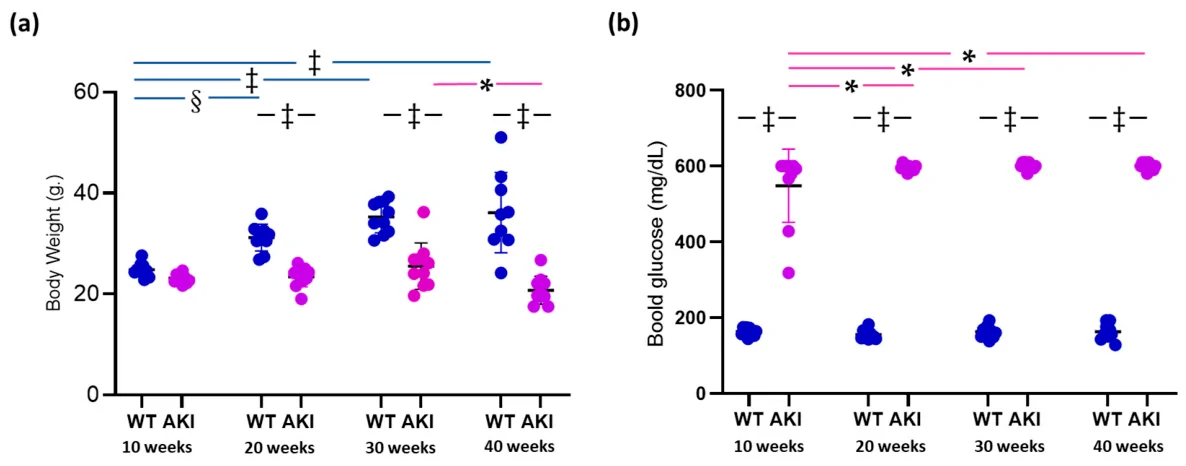
(**a**) Body weight (B.W., g.) at 10, 20, 30 and 40 weeks of age in Akita (n = 8–10 for each timepoint) and WT mice (n = 8–10 for each timepoint). Data are presented as a dot plot, reporting mean ± SD. Statistical analysis was performed by two-way ANOVA, followed by Bonferroni’s post hoc test. *: *p* < 0.05; §: *p* < 0.01; ‡: *p* < 0.0001. (**b**) Non-fasting blood glucose levels (mg/dL) at 10, 20, 30 and 40 weeks of age in Akita and WT mice. Data are presented as a dot plot, reporting mean ± SD. Statistical analysis was performed by two-way ANOVA, followed by Bonferroni’s post hoc test. *: *p* < 0.05; ‡: *p* < 0.0001. Black horizontal lines indicate the differences between Akita and WT, blue horizontal lines indicate the differences within WT, and fuchsia horizontal lines indicate the differences within Akita mice.

**Figure 2:**
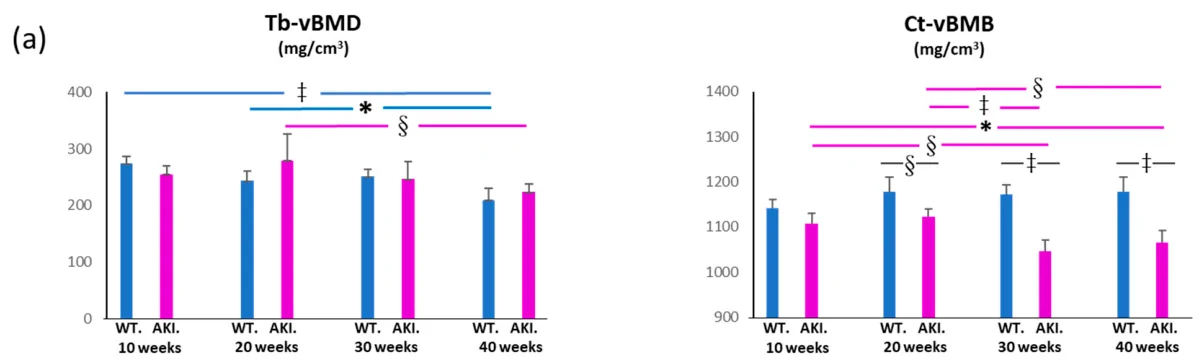

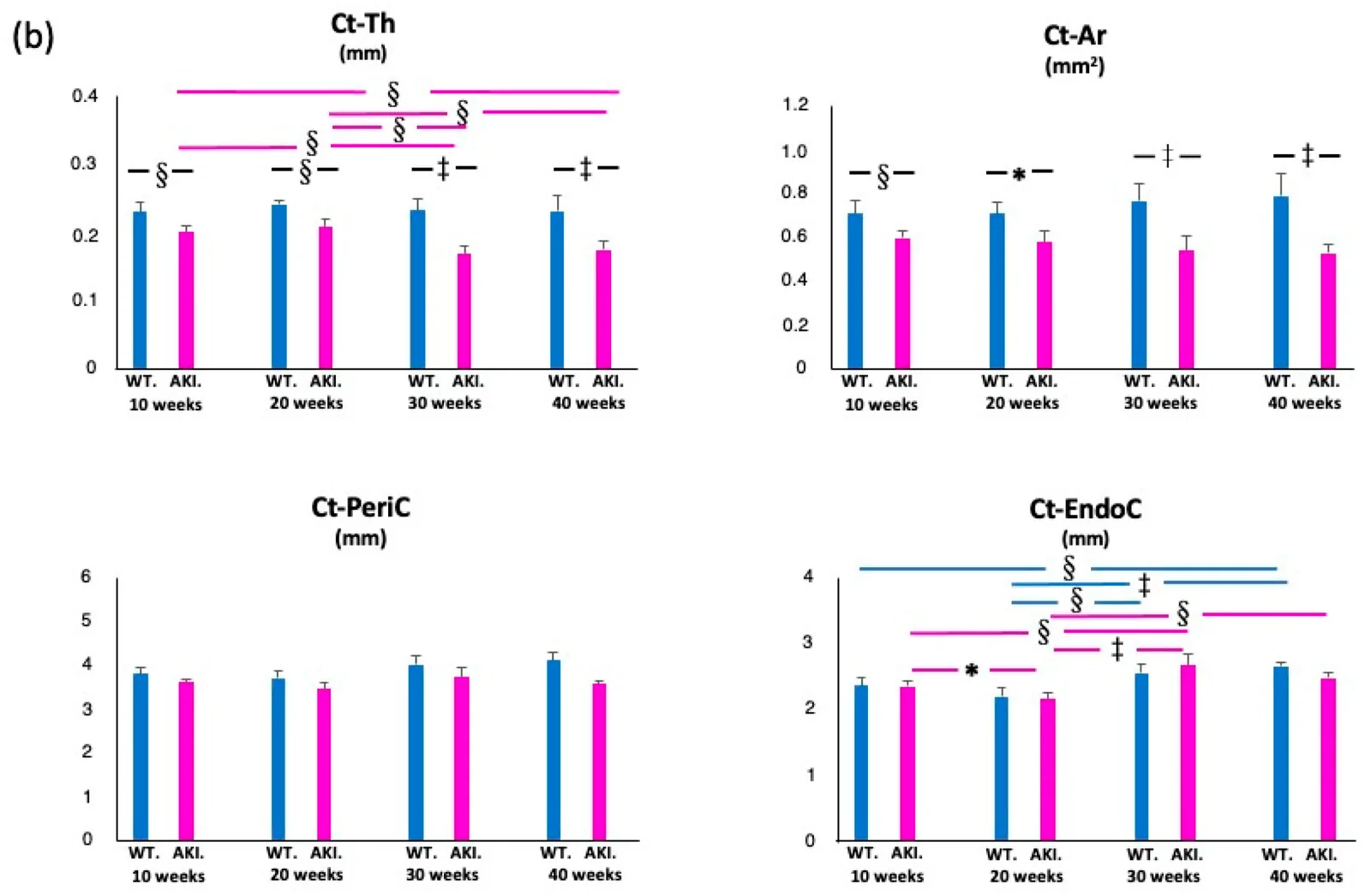
Bone parameters measured by pQCT in the tibiae of Akita and WT mice at different ages. (**a**) Tb.vBMD: trabecular volumetric bone mineral density, Ct.vBMD: cortical volumetric bone mineral density. (**b**) Ct-Th: cortical thickness, Ct.Ar: cortical area, Ct-PeriC: periosteal circumference, Ct-EndoC: endosteal circumference. Tb.vBMD was measured at the proximal metaphysis of the tibia; Ct.vBMD was measured at the diaphyseal midshaft (n = 6–8 for each timepoint). Data are presented as mean ± SD. Statistical analysis was performed by two-way ANOVA, followed by Bonferroni’s post hoc test. *: *p* < 0.05; §: *p* < 0.01; ‡: *p* < 0.0001. Black horizontal lines indicate the differences between Akita and WT, blue horizontal lines indicate the differences within WT, and fuchsia horizontal lines indicate the differences within Akita mice.

**Figure 3:**
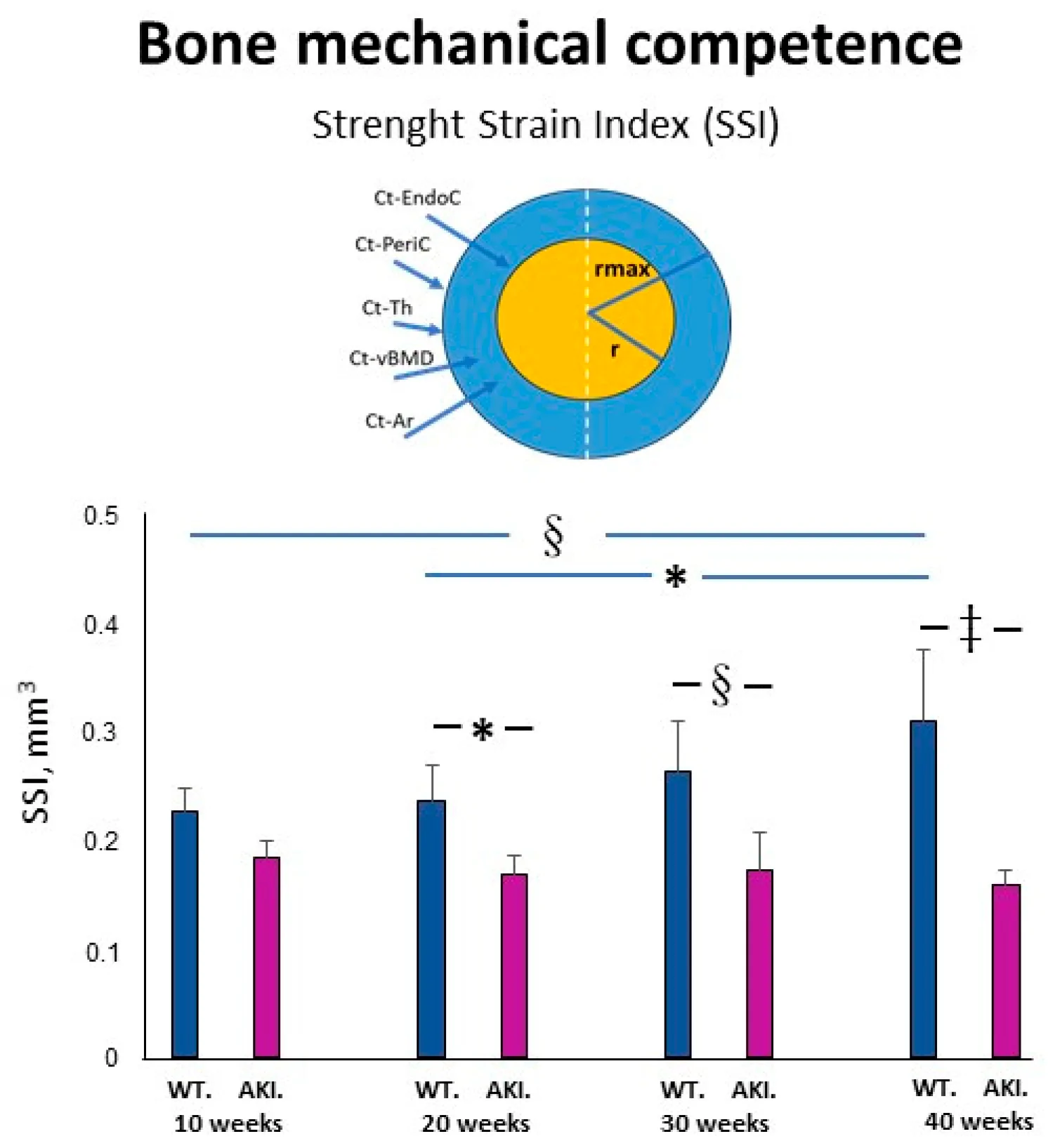
Polar strength strain index (SSI), which integrates the bone geometric parameters with volumetric bone mineral density (showed schematically in the picture. Section of diaphysis: yellow = medullary cavity; blue = cortex; r = radius; rmax = maximum radius; dashed line = diameter) at 10, 20, 30 and 40 weeks of age in Akita (n = 5–6 for each timepoint) and WT (n = 5–6 for each timepoint) mice. Data are presented as mean ± SEM. Statistical analysis was performed by two-way ANOVA, followed by Bonferroni’s post hoc test. *: *p* < 0.05; §: *p* < 0.01; ‡: *p* < 0.0001. Black horizontal lines indicate the differences between Akita and WT, blue horizontal lines indicate the differences within WT.

**Figure 4:**
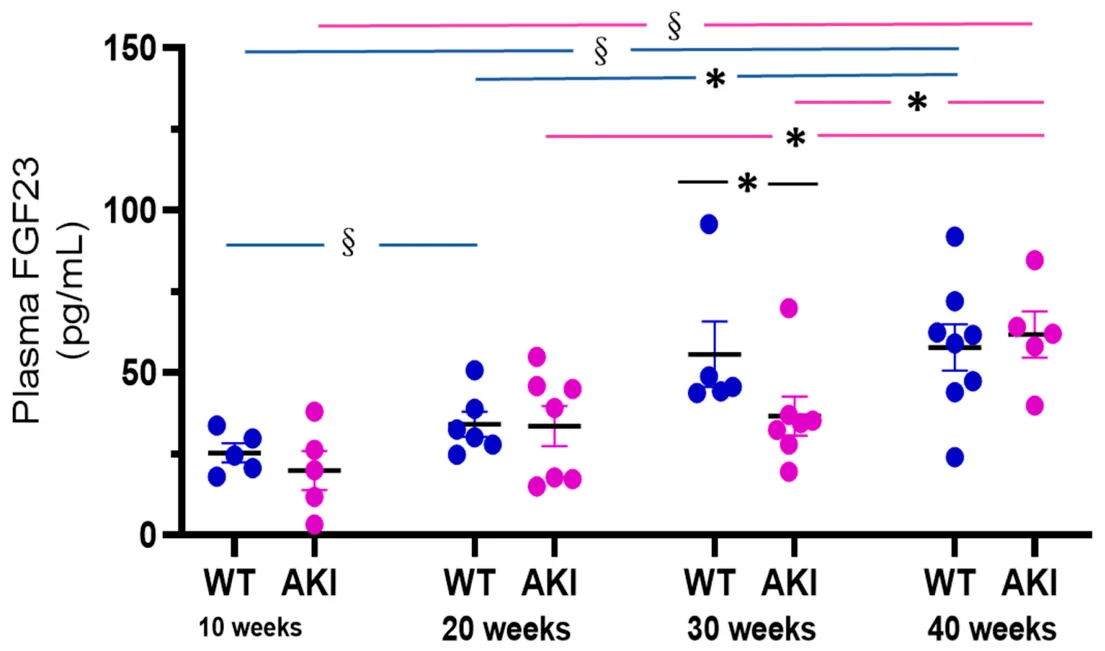
Plasma fibroblast growth factor 23 (pg/mL) at 10, 20, 30 and 40 weeks of age in Akita (n = 5–7 for each timepoint) and WT (n = 5–8 for each timepoint) mice. Data are presented as a dot plot, reporting mean ± SD. Statistical analysis was performed by two-way ANOVA, followed by Bonferroni’s post hoc test. *: *p* < 0.05; §: *p* < 0.01. Black horizontal lines indicate the differences between Akita and WT, blue horizontal lines indicate the differences within WT, and fuchsia horizontal lines indicate the differences within Akita mice.

**Figure 5:**
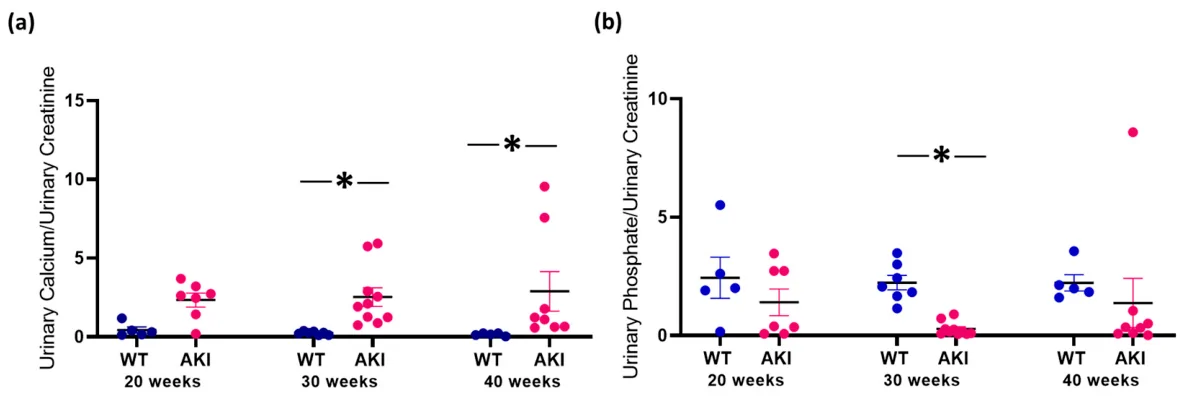
(**a**) Urinary calcium-to-creatinine ratio and (**b**) urinary phosphate-to-creatinine ratio at 20, 30 and 40 weeks of age in Akita (n = 7–10 for each timepoint) and WT (n = 5–7 for each timepoint) mice. Data are presented as a dot plot, reporting mean ± SD. Statistical analysis was performed by two-way ANOVA, followed by Bonferroni’s post hoc test. *: *p* < 0.05.

**Figure 6:**
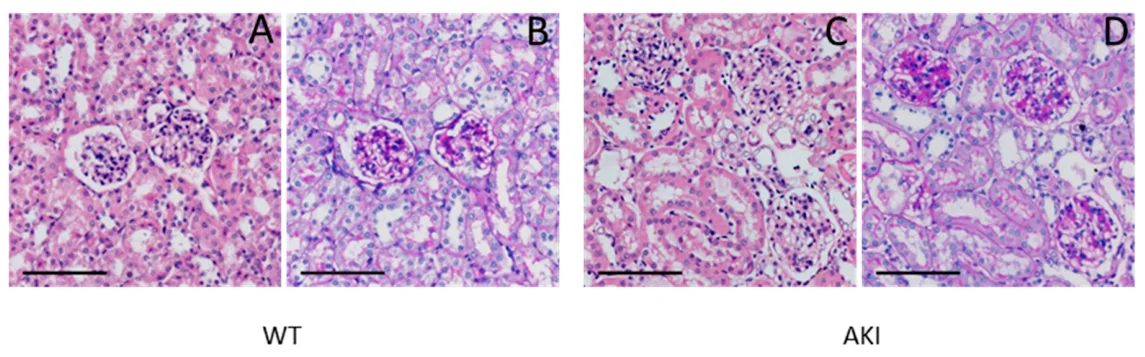
Representative microphotographs of renal tissue from 40-week-old WT ((**A**,**B**), n = 5) and Akita ((**C**,**D**), n = 5) mice. Hematoxylin-eosin stain (**A**,**C**). Periodic acid–Schiff stain (**B**,**D**). Scale bar 100 μm.

**Figure 7:**
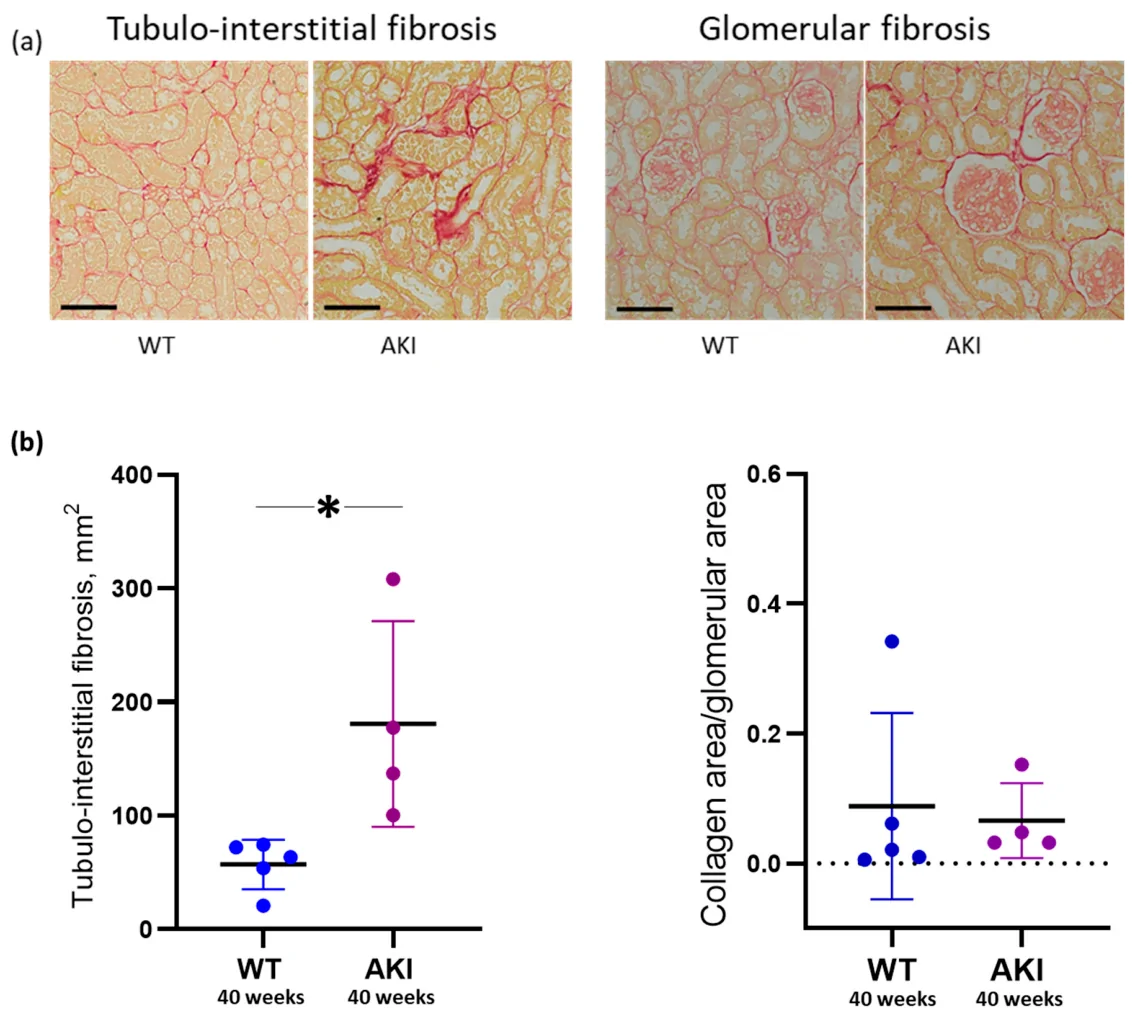
(**a**): Representative microphotographs of renal tubulo-interstitial and glomerular fibrosis in Akita (n = 4) and WT (n = 5) mice (scale bar 100 μm). (**b**): Quantification of tubulo-interstitial and glomerular fibrosis in Akita and WT mice. Data are presented as a dot plot, reporting mean  ± SD. Statistical analysis was performed using the Mann–Whitney test. *: *p* < 0.05.

**Table 1:** Plasma creatinine (mg/dL) and phosphate (mg/dL) at 10, 20, 30 and 40 weeks of age in Akita (n = 5–6 for each timepoint) and WT (n = 5–6 for each timepoint) mice.

	10 Weeks		20 Weeks		30 Weeks		40 Weeks	
Plasma	WT	AKI	WT	AKI	WT	AKI	WT	AKI
Creatinine (mg/dL)	0.150 ± 0.03	0.170 ± 0.04	0.270 ± 0.04	0.160 ± 0.06	0.128 ± 0.01	0.104 ± 0.01	0.129 ± 0.01	0.077 ± 0.02
Phosphate (mg/dL)	6.97 ± 0.47	7.55 ± 0.42	5.58 ± 0.38	6.28 ± 0.72	4.91 ± 0.68 *	5.79 ± 1.00	6.57 ± 0.33	5.16 ± 0.43 §

Data are presented as mean ± SEM. Statistical analysis was performed by two-way ANOVA, followed by Bonferroni’s post hoc test. *: *p* < 0.05 vs. WT at 10 weeks of age; §: *p* < 0.05 vs. AKI at 10 weeks of age.

## Data Availability

The original contributions presented in this study are included in the article. Further inquiries can be directed to the corresponding author.

## Abbreviations

The following abbreviations are used in this manuscript:

Ct-Ar	cortical area
Ct-EndoC	endosteal circumference
Ct-PeriC	periosteal circumference
Ct-Th	cortical thickness
Ct-vBMD	cortical volumetric bone mineral density
CTX-1	C-terminal telopeptide of type I collagen
FGF23	fibroblast growth factor 23
maxr	maximum radius
pQCT	peripheral quantitative computed tomography
r	radius
SSI	strength strain index
STZ	streptozotocin
T1DM	type 1 diabetes mellitus
T2DM	type 2 diabetes mellitus
Tb.vBMD	trabecular volumetric bone mineral density
Wnt	Wingless Integrase-1
